# Study on the Effect of Cold Deformation and Heat Treatment on the Properties of Cu-Ag Alloy Wire

**DOI:** 10.3390/mi14081635

**Published:** 2023-08-19

**Authors:** Xuefeng Wu, Hewei Jia, Junling Fan, Jun Cao, Chenghao Su

**Affiliations:** 1 School of Mechanical and Power Engineering, Henan Polytechnic University, Jiaozuo 454000, China; 212105020051@home.hpu.edu.cn (H.J.); cavan@hpu.edu.cn (J.C.); 212005020029@home.hpu.edu.cn (C.S.); 2 Chemical and Environmental Engineering, Jiaozuo University, Jiaozuo 454003, China; jzufanjunling@163.com

**Keywords:** multi-pass, pulling annealing, tensile strength, elongation, resistivity

## Abstract

The effects of various drawing parameters and annealing processes on the structure and properties of Cu-Ag wires, containing 1 wt% silver, were investigated using specialized equipment including fine wire-drawing machines, very fine wire-drawing machines, heat treatment equipment, tensile testing machines, microcomputer-controlled electronic universal testers, resistance testers, and scanning electron microscopes. The results revealed that continuous drawing of Cu-1%Ag alloy wires led to elongation of the grains, resulting in a uniform and tightly fibrous microstructure. Moreover, the tensile strength of the alloy wire increased from 670 MPa to 783.9 MPa after a single pass with a deformation of 14%. Subsequently, when the wire was drawn at a speed of 500 m/min, the tensile strength further increased to 820.1 MPa. After annealing the Փ0.08 mm Cu-1% Ag alloy wire, an increase in annealing temperature up to 500 °C resulted in the wire’s tensile strength decreasing from 820.1 MPa to 377.5 MPa. Simultaneously, the elongation increased from 1.94% to 15.21%, and the resistivity decreased from 1.931 × 10^−8^ Ω·m to 1.723 × 10^−8^ Ω·m. Additionally, when annealing was conducted at a rate of 80 m/min, the wire resistivity dropped to 1.635 × 10^−8^ Ω·m.

## 1. Introduction

The rapidly expanding microelectronics industry has presented significant challenges in chip packaging technology and materials. These challenges encompass a range of requirements, such as achieving enhanced levels of hermeticity and integration, improving heat dissipation capabilities, and ensuring the reliability of packaged chips. As the industry continues to evolve, meeting these demands has become crucial for advancements in microelectronic devices and systems [1,2]. Wire bonding is widely recognized as the most popular chip interconnection technology in microelectronic packaging due to its mature process, excellent bonding performance, high reliability, and cost-effectiveness [3,4,5,6]. The bonding wire, serving as the core material of the package, plays a crucial role in connecting the pins and silicon wafers, as well as transmitting electrical signals. Currently, there is a demand for finer wire diameters and higher strength in bonding wire technology. Additionally, it is essential to maintain good plasticity, stable chemical properties, and high conductivity. In comparison to gold- and silver-based alloy wires, copper–silver alloy wires offer high strength, high hardness, and cost-effectiveness, while still preserving excellent electrical and thermal conductivity [7,8,9,10,11].

With the continuous development of integrated circuits and chips, there is a growing demand for copper–silver alloys with high strength and high conductivity. These alloys are sought after due to their ability to meet the requirements of modern-day technology and provide enhanced performance in various applications. The Cu-1 wt%Ag alloy with 98% cold-rolled depression prepared by Jianlan Chen in combination with intermediate heat treatment obtained a comprehensive tensile strength of 956 MPa and a relative electrical conductivity of 72% [12]; Lin-Sheng Tang used vacuum casting to prepare Cu-4.5 wt%Ag alloy, through the “casting-peak aging-cold drawing” process, the strength of copper–silver alloy wire reached 830 MPa, an electrical conductivity of 85% IACS [13]. Yanjun Zhou et al. prepared Cu-3.5Ag alloy using “solid solution-ageing-cold deformation” and “solid solution-cold deformation-ageing” processes, which resulted in a tensile strength of 417.2 MPa and a conductivity of up to 95.5% IACS [14]. Kexing Song et al. investigated the dynamic deformation behavior of Cu-20Ag alloys and concluded that the increase in strain rate promoted Ag precipitation, hindered the movement of dislocations, and increased the stress and yield strength of Cu-Ag alloys [15]. By comparing and analyzing the properties of Cu-Ag alloys with different compositions, Fei Cao et al. provide references and ideas for the preparation of ultra-high strength, high conductivity and high Ag content Cu-1 wt%Ag alloys [16]. Mingwang Xie used multi-pass drawing combined with intermediate annealing process to prepare a high-strength, high-conductivity Cu-20Ag alloy with a strength of 1175 MPa and an electrical conductivity of 80.4% IACS [17]. Varol Temel et al. used a new Cu-Ag alloy prepared by chemical plating and hot pressing and found that the hardness and density of the material increased with the proportion of silver-plated copper powder in the copper powder [18]; Choi Eun-Ae et al. designed a thermomechanical treatment process for subeutectic Cu-12Ag alloys to obtain their tensile strength up to 1189 MPa and electrical conductivity up to 71.9% IACS [19]. In Cu-Ag alloy wires, the electrical conductivity of Ag content in 3–28% is relatively poor, and Ag content of 0.1% will lead to poor tensile strength [20], so the study of Cu-1 wt%Ag alloy wires is of great significance for the preparation of high-performance bonding wires. Most of the previous research has focused on copper–silver alloy wires with diameters exceeding 0.1 mm. However, there has been relatively limited investigation into microfine copper–silver alloy wires with diameters below 0.1 mm. This paper aims to fill this research gap by focusing on studying copper–silver alloy wires with smaller diameters. The objective is to explore the influence of various drawing parameters and annealing processes on the properties and microstructure of these wires.

## 2. Test Materials and Methods

### 2.1. Test Materials and Equipment

The experiments conducted in this study utilized Cu-1 wt%Ag wire with a silver content of 1 wt%. The wire initially had a diameter of 0.25 mm in its unannealed state. The initial parameters of the wire were as follows: a tensile strength of 670 MPa, an elongation of 1.86%, and a resistivity of 1.922 × 10^−8^ Ω·m. The necessary equipment for conducting these tests is listed in Table 1.

**Figure 1:**
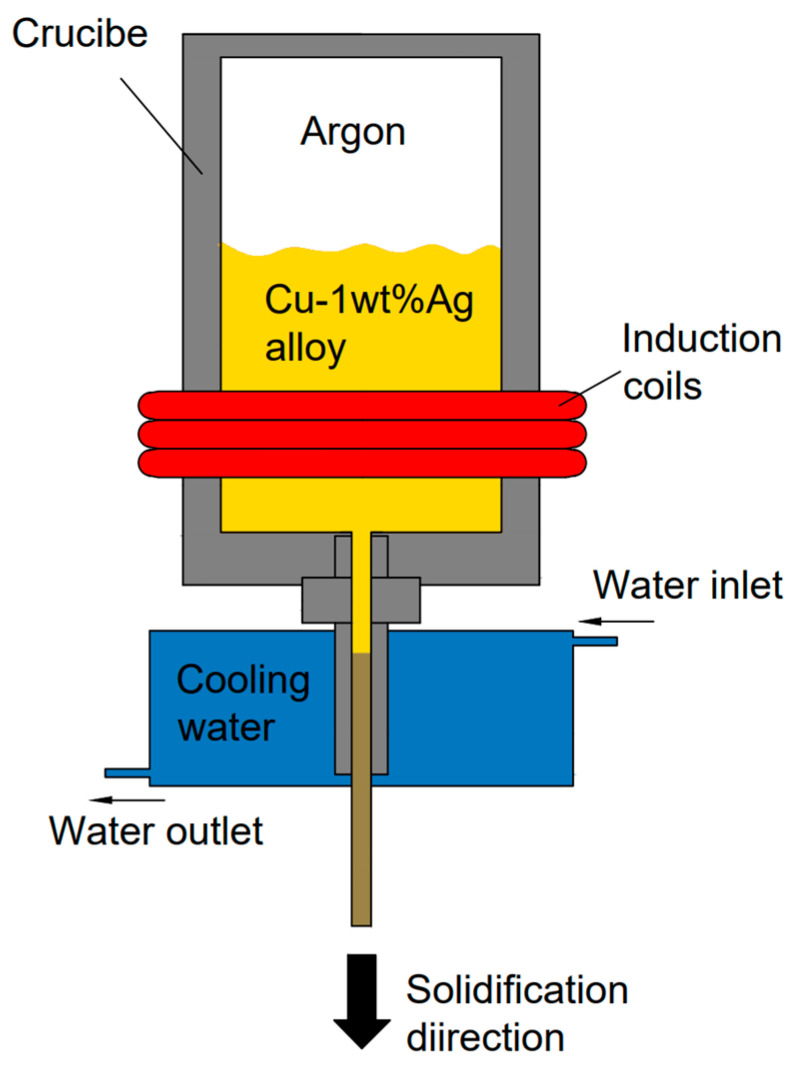
Continuous casting equipment diagram.

The test material is an 8.0 mm diameter Cu-1Ag alloy rod prepared by vertical leaded vacuum melting directional solidification continuous casting machine. The 8.0 mm diameter alloy rod was drawn to a 3.0 mm diameter by the large-wire-diameter drawing machine, then to a 0.9 mm diameter by the medium-wire-diameter drawing machine with multiple dies, and finally processed to a 0.25 mm diameter alloy wire by the small-wire-diameter drawing machine with multiple dies. The principle of the continuous casting machine is shown in Figure 1. The continuous casting parameters employed are as follows: the melting temperature is 1250 °C, the cooling temperature is 25 °C, the traction speed is 80 mm/min, the traction time is 0.5 s, and the stopping time is 0.5 s.

**Figure 2:**
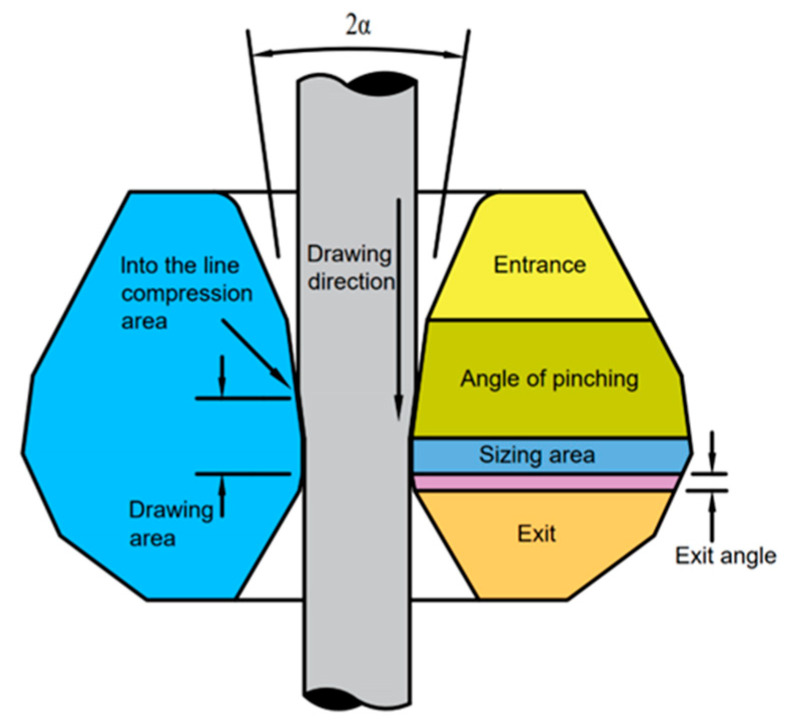
Drawing die.

The wire-drawing die primarily consists of a die sleeve and a die core. The die sleeve serves to provide force support for the core during the drawing process of high-strength wire, preventing the core from rupturing. The conventional dimensions of the die sleeve are Փ25 × 8 mm. On the other hand, the die core is comprised of four components: the entrance area, compression area, sizing area, and exit area. Natural diamond is the material used for the die core. The wire-drawing die is depicted in Figure 2. The entrance area is typically designed with a circular arc, allowing for easy entry of the wire into the working area. The angle of the circular arc is usually set to 60°. As the wire material progresses through the compression area, the drawing fluid creates a lubricating layer on the surface of the wire material, initiating plastic deformation. In this region, selecting an appropriate working cone angle, denoted as 2α, is crucial for determining the magnitude of the drawing force. The choice of working cone angle should follow the principle that, for a higher compression ratio and harder drawing material, a smaller working cone angle is preferred. For copper wire drawing, a cone angle of 16°–18° is commonly employed. The primary purpose of the sizing area is to achieve the final dimensions of the wire material. Its length is generally 0.5–1.0 times the diameter of the wire. The exit area serves as the final section where the material exits the die hole. It plays a role in protecting the sizing zone and the wire material, with an exit angle ranging from 45° to 65°.

In the wire-drawing process, the amount of deformation is a crucial factor that significantly affects the wire’s performance. Excessive deformation can result in higher deformation resistance, making the process more difficult and increasing the likelihood of wire breakage during drawing. Furthermore, it can also lead to higher hardness of the wire after drawing. On the other hand, insufficient deformation will require a greater number of drawing passes, reducing drawing efficiency. In this specific test, the wire was initially drawn to a size of 0.25 mm. Subsequently, it was further drawn to 0.135 mm using different drawing passes and a single-pass strain. Finally, the wire was drawn to 0.08 mm using different drawing rates, with varying levels of strain as indicated in Table 2. The drawing strain can be calculated using the following formula:

(1) $$\varepsilon =\ln(l_{1}/l_{0})$$

l_0_ is the length of the wire before drawing (mm);

l_1_ is the length of the wire after drawing (mm).

According to the principle of constant volume, and considering that the cross-section of the test wire is circular, the formula can be deformed to

(2) $$\varepsilon =\ln\left(\frac{A_{0}}{A_{1}}\right)=2\ln\left(\frac{D_{0}}{D_{1}}\right)$$

A_0_ is the cross-sectional area of the wire before drawing (mm^2^);

A_1_ is the cross-sectional area of the wire after drawing (mm^2^);

D_0_ is the diameter of the wire before drawing (mm);

D_1_ is the diameter of the wire after drawing (mm).

**Figure 3:**
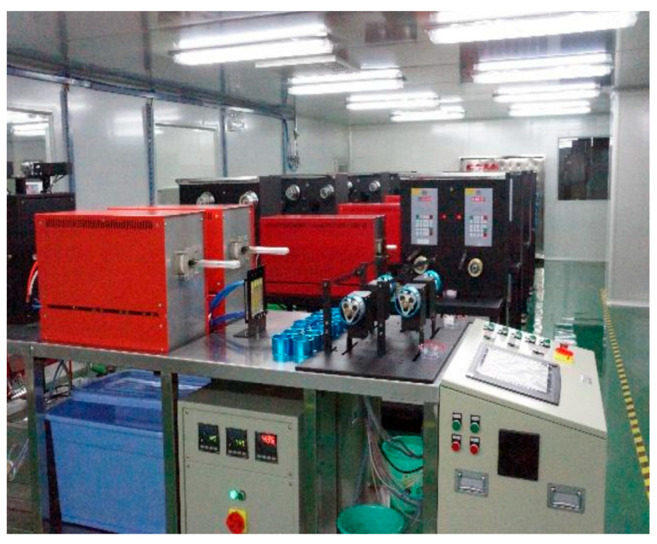
Continuous heat treatment equipment.

In the heat treatment test, a diameter of 0.08 mm Cu-1Ag alloy wire in the SD-40 continuous heat treatment equipment for heat treatment, heating for electric heating, annealing tube material for quartz glass, an annealing tube length of 1200 mm, a temperature fluctuation range of 2 °C, heat treatment equipment using an angular displacement sensor to control the tension of the wire, and a tension range of 0.01–0.10 N were used. The annealing equipment is shown in Figure 3. In the different annealing rate tests, the annealing rate refers to the speed at which the alloy wire passes through the annealing tube and is the rate at which the wire is wound during the annealing heat treatment.

### 2.2. Test Method

The initial Cu-1 wt%Ag wires with a diameter of 0.25 mm underwent multi-pass drawing tests to achieve alloy wires with a diameter of 0.135 mm. The drawing process followed the test scheme presented in Table 3, with single-pass drawing amounts of 18%, 14%, and 10%, respectively, at a drawing speed of 500 m/min. Sample intercepts were taken at each pass to test the tensile strength, elongation, and resistivity of the alloy wires. Furthermore, metallographic samples were obtained from the final passes to observe the effect of single-pass deformation on the microstructure of Cu-1 wt%Ag bonded wire.

For subsequent tests, the wire that underwent a single-pass deformation of 14% was selected. The test procedure is outlined in Table 4. In this test, the Փ0.135 mm alloy wire was subjected to three different drawing speeds: 300 m/min, 500 m/min, and 700 m/min. Each pass involved a single-pass drawing of 15%, resulting in a final alloy wire with a diameter of 0.08 mm. Samples were intercepted from each pass to measure the tensile strength, elongation, and resistivity of the wire. Additionally, metallographic samples were obtained from the final pass to observe the impact of the drawing rate on the microstructure of the Cu-1 wt%Ag bonded wire.

In the above test, the wire with a drawing rate of 500 m/min was selected for annealing heat treatment according to the test procedure outlined in Table 5. The 0.08 mm wire was annealed at temperatures of 250 °C, 300 °C, 350 °C, 400 °C, 450 °C, 500 °C, and 550 °C, respectively, with an annealing rate of 100 m/min. Samples were collected after the annealing process to measure the tensile strength, elongation, and resistivity of the wire. The mechanical and electrical properties of the Cu-1 wt%Ag bonded wires were studied at different annealing temperatures. Metallographic samples were also taken from the wires that underwent annealing at different temperatures. These samples were observed using scanning electron microscopy (SEM) to analyze the evolution of the material microstructure under changing temperature conditions.

The annealing rate test was conducted using a wire material with a diameter of 0.08 mm and a drawing speed of 500 m/min. The test followed the protocol outlined in Table 5, which included annealing rates of 20 m/min, 40 m/min, 60 m/min, 80 m/min, 100 m/min, and 120 m/min. The annealing temperature for the heat treatment test was consistently maintained at 500 °C. After each stage of the test, samples were carefully collected and subjected to thorough analysis, measuring key properties such as tensile strength, elongation, and resistivity. This comprehensive examination allowed for a detailed investigation into how the material’s properties evolve at various annealing rates.

The SEM samples were prepared as follows: The intercepted samples were put into the molds and poured with epoxy resin to solidify naturally. After that, the sanding operation was performed on the polishing machine using 600#, 800#, 1000#, 1200#, and 2000#SiC sandpaper, and the surface of the sandpaper was rinsed with water stream throughout. After each stage of polishing, the specimen was rotated by 90°, and each stage of polishing was subject to the absence of the last scratch on the surface. After the above grinding steps, the surface of the specimen was free of obvious scratches. The specimen underwent rough and fine polishing using 1.0 μm and 0.5 μm diamond suspension sprays, respectively. Following the fine polishing, the specimen’s surface exhibited a smooth texture without any visible scratches when observed under a microscope. A corrosion solution was prepared by mixing 5 g of copper chloride with 100 mL of ammonia solution. This ready-to-use corrosion solution was applied for a duration of 5–10 s. Subsequently, the specimen was thoroughly cleaned using distilled water and anhydrous ethanol, followed by gentle blowing to ensure complete dryness. The cleaned surface of the specimen was then coated with a layer of gold before being placed under a scanning electron microscope. Various magnifications were used to observe the microstructure of the specimen, allowing for the analysis of variations and distinctions in microstructure at different levels of deformation, drawing rates, and annealing conditions.

## 3. Results

### 3.1. Effect of Different Deformation on the Microstructure of Cu-1 wt%Ag Wire

**Figure 4:**
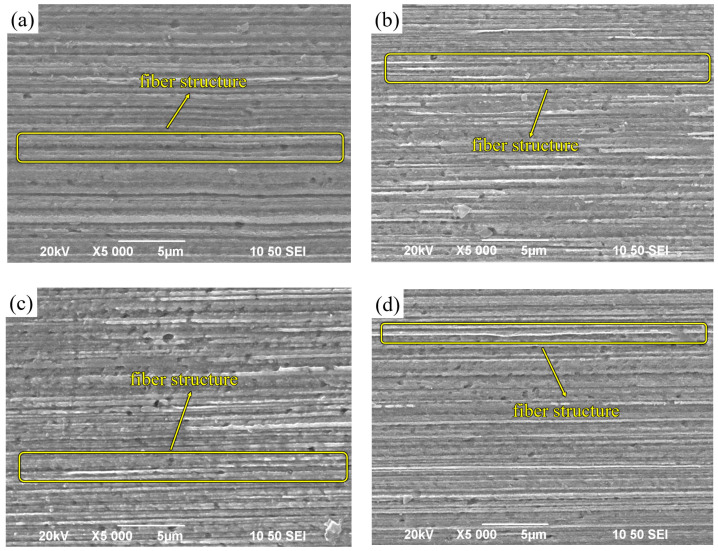
Longitudinal microstructure of Cu−1 wt%Ag wires after different single-pass deformation tests ((**a**) initial wire (**b**) 18% (**c**) 14% (**d**) 10%).

Figure 4 presents SEM images of a Cu-1 wt%Ag wire with a diameter of 0.25 mm after undergoing three different single-pass deformations during the drawing process. In Figure 4a, the initial microstructure of the wire appears striped, clearly displaying the presence of grain boundaries. Figure 4b–d depict the wire after being drawn with varying single-pass deformations. In each case, the grains are further elongated in the stretching direction, resulting in a fibrous microstructure that is neatly arranged along the drawing direction. Additionally, the interfaces between different phases become straighter, and the overall distribution becomes more uniform and compact. It is worth noting that there are no significant differences in the fibrous grain morphology among the wires drawn using the three different deformation schemes. However, the scheme involving a single-pass deformation of 10% exhibits a more uniform grain size distribution. According to the Cu-1 wt%Ag phase diagram, the solid solubility of Ag in Cu at room temperature is only 0.1 wt%. In the as-cast sample of Cu-1 wt%Ag, a portion of Ag is dispersed within the Cu matrix. During the drawing and deformation process, the grains gradually undergo breakage and refinement, leading to the Ag grains being drawn into a fibrous structure distributed along the axial direction.

### 3.2. Effect of Different Deformation on the Performance of Cu-1 wt%Ag Wire

The drawing process induces lattice distortion, leading to the formation of an elastic stress field around dislocations. Consequently, Ag atoms in the vicinity of dislocations tend to migrate, thereby generating additional stress around dislocations and contributing to the improved strength of the Cu-1 wt%Ag bonded alloy wire. According to metal-electron theory, the disruption of the crystal lattice integrity results in scattering, which is the fundamental cause of resistance in metals. The lattice distortion and dislocation entanglement occurring during the drawing process disrupt the crystal lattice integrity, thereby enhancing the scattering effect on metal electrons. During continuous multi-pass drawing, interfacial scattering primarily influences the resistivity of the Cu-1 wt%Ag wire. This is because the average diameter of the fibrous grains decreases, leading to an increase in material resistivity. Additionally, as the strain increases, the solid solution of Ag atoms within the copper matrix also increases. This causes certain Ag atoms to dissolve into the copper matrix, weakening the scattering effect at the phase interface. Consequently, the resistivity of the wire material decreases during the drawing process.

**Figure 5:**
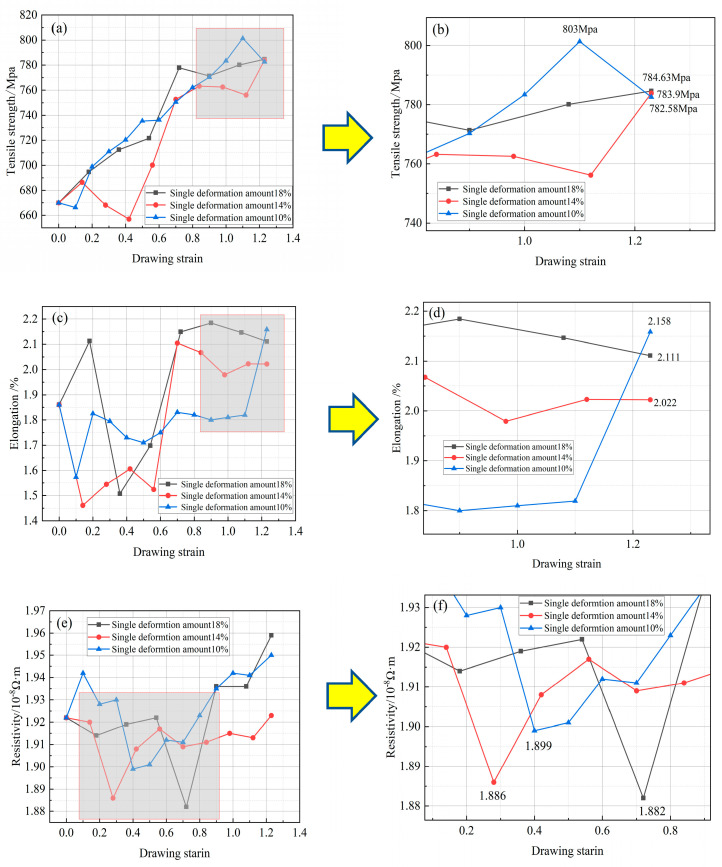
Mechanical and electrical properties of Cu−1 wt%Ag wire at different deformation amounts ((**a**,**b**) tensile strength; (**c**,**d**) elongation; (**e**,**f**) resistivity).

Based on the variation curves of mechanical properties in Figure 5a–d, the tensile strength of the Cu-1 wt%Ag wire after drawing is similar for different deformation levels. The tensile strengths are approximately 784.63 MPa, 783.9 MPa, and 782.58 MPa, respectively. Overall, the tensile strengths of all three samples increase with drawing. In the case of a single deformation of 18%, the material’s tensile strength reaches 777.86 MPa at a strain of around 0.7. After this point, the tensile strength remains stable, while the elongation increases from 1.8% to 2.022%. For a single deformation of 14%, the material’s tensile strength reaches 762.3 MPa at a strain of about 0.7. It then stabilizes and further increases to 783.9 MPa at a strain of 1.23. The elongation also rises from 1.8% to 2.111%. With a single deformation of 10%, the tensile strength of the Cu-1 wt%Ag wire reaches a maximum value of 803 MPa at a strain of 1.1, representing a 20% increase compared to the initial wire. Further deformation reduces the tensile strength to 783.9 MPa, while the elongation increases from 1.8% to 2.158%. This results in an approximately 17% increase in tensile strength compared to the initial sample. During multiple drawing passes, the wire structure becomes fibrillated, increasing the deformation stress and filament strength. The solid dissolution of Ag atoms in the matrix causes localized distortion points. This distortion increases the resistance to crystal slip, making deformation more difficult. As plastic deformation progresses, internal defects in the wire multiply, dislocations rapidly proliferate, and dislocation density continuously increases. The grains elongate and deform along the drawing direction, leading to a continuous increase in tensile strength.

As strain increases, the interface between the eutectic and Cu matrix absorbs the dislocation substructure. Subsequently, the dislocation migrates to the interface area between the Cu solid solution and eutectic fiber. The overall dislocation density decreases, and the level of interfacial strengthening reaches a higher point. As a result, the material properties change more gradually.

Figure 5e,f illustrate the resistance change curves of Cu-1 wt%Ag wire at different deformation levels. In the case of 18% deformation, the resistivity exhibited a relatively smooth pattern during the drawing process. However, a significant decrease in resistivity (to 1.882 × 10^−8^ Ω·m) occurred at a strain of approximately 0.7, followed by a sustained growth trend. The resistivity reached its minimum values at a strain of 0.28 and 0.4 (1.886 × 10^−8^ Ω·m and 1.899 × 10^−8^ Ω·m, respectively) when the deformation level was reduced to 14% and 10%. Subsequently, the resistivity slowly increased and remained stable.

### 3.3. Effect of Different Drawing Rates on the Microstructure of Cu-1 wt%Ag Wire

**Figure 6:**
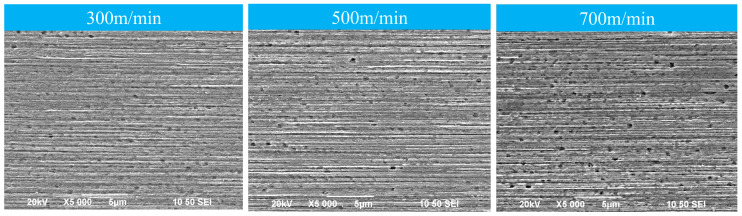
Longitudinal microstructure of Cu−1 wt%Ag wires after different drawing rate tests.

Comparison of Figure 4 and Figure 6 shows that the microstructure of the longitudinal section of the alloy wire tends to be increasingly fibrous with the continuation of drawing, although the effect of different drawing rates on the microstructure of the material is not obvious, but due to the continuous deformation of multiple passes, the grain refinement and torsion deformation are extremely serious, and the spacing of the fibrous tissue layers decreases gradually, and Figure 6 exhibits an obvious fiber-like structure. The fibrous organization becomes increasingly compact because, when the fibrillation of the material is completed, the increase in strain does not change the morphology of the fibers but reduces the fiber grain diameter.

### 3.4. Effect of Different Drawing Rates on the Properties of Cu-1 wt%Ag Wire

**Figure 7:**
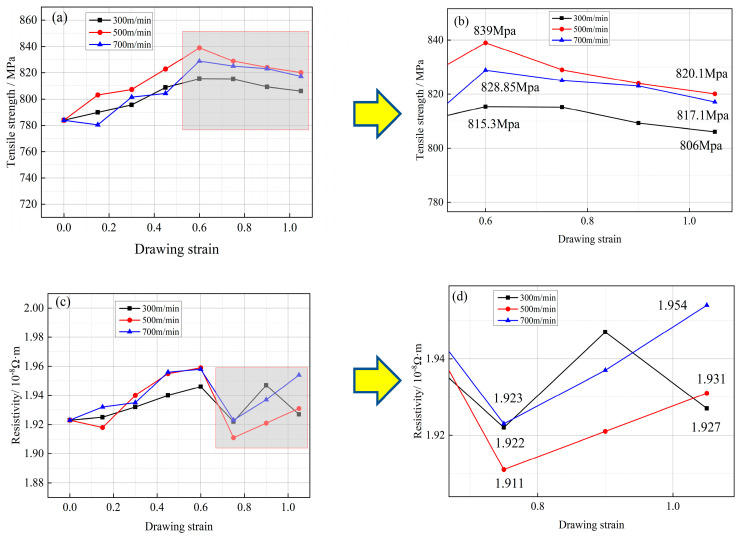
Mechanical and electrical properties of Cu−1 wt%Ag wire at different drawing rates ((**a**,**b**) tensile strength; (**c**,**d**) resistivity).

The tensile strength of Cu-1 wt%Ag wire increased from 783.9 MPa to 806 MPa, 820.1 MPa, and 817.1 MPa after drawing at rates of 300 m/min, 500 m/min, and 700 m/min, respectively. Figure 7a,b depict the changes in tensile strength of Cu-1 wt%Ag wire during the drawing process at different rates. The variation in tensile strength was similar for different drawing rates: it exhibited an increasing trend followed by a plateau as the strain increased, reaching its peak at a strain of 0.6 in all cases (815.3 MPa, 839 MPa, and 828.85 MPa).

Figure 7c,d demonstrate the change in resistivity of Cu-1 wt%Ag material during the wire-drawing process at various speeds. The resistivity increases from 1.923 × 10^−8^ Ω·m to 1.927 × 10^−8^ Ω·m, 1.931 × 10^−8^ Ω·m, and 1.954 × 10^−8^ Ω·m, respectively. At a drawing speed of 300 m/min, the resistivity change exhibits a relatively smooth trend, whereas at 500 m/min, it shows significant fluctuations. Furthermore, all scenarios experience a substantial decrease in resistivity at a strain of 0.75, reaching the lowest values of 1.922 × 10^−8^ Ω·m, 1.911 × 10^−8^ Ω·m, and 1.923 × 10^−8^ Ω·m, respectively. During the wire-drawing process, as plastic deformation continues, the crystal lattice structure becomes distorted. This distortion leads to the generation of numerous dislocations, vacancies, and other defects at the boundaries between individual grains. These defects have a scattering effect on the free electrons present in the material, causing an increase in resistivity. Additionally, the Cu-1 wt%Ag material contains second-phase Ag particles. These Ag particles interact with the dislocations generated during plastic deformation. Such interactions further contribute to an increase in resistivity within the wires. The presence of these second-phase particles introduces additional obstacles for the movement of free electrons, leading to an overall increase in resistivity. Huadong Fu et al. pointed out that the refinement of fibers due to plastic deformation leads to an increase in interfaces with higher tensile strain, resulting in a decrease in electrical conductivity [21].

After being drawn at three different rates, the Cu-1Ag wire exhibits minimal changes in tensile strength. At this stage, significant work hardening occurs within the Cu-1Ag wire. The presence of Ag increases the concentration limits of dislocations and subgrain boundaries in the Cu solid solution, resulting in a higher degree of hardening compared to pure copper wire [22]. In the initial stages of drawing, cold deformation results in an increased dislocation density. Work hardening and dislocation strengthening contribute to a rise in tensile strength. As the deformation progresses to a certain extent, the movement of dislocations becomes more difficult, leading to saturation of dislocation density and minimal changes in grain size. Consequently, the effect on tensile strength becomes less pronounced. Zhu Xiao et al. showed that, with the continuation of the drawing, in the stage of large deformation, the tensile strength of Cu-Ag alloys will slowly increase [23]. As the strain increases, the solid solubility of Ag within the Cu matrix also increases. When the strain reaches a certain threshold, a portion of the secondary Ag phase undergoes back dissolution. This results in a decrease in the area of the Ag/Cu phase boundary and subsequently reduces the scattering effect on electrons. Consequently, the wire resistivity decreases when the strain reaches 0.75.

The mechanical properties, electrical properties and microstructure of the alloy wires after drawing with different deformation behaviors have certain trends, which will be compared with the data of the heat-treated alloy wires and the analysis of the organization evolution law to find the process parameters for the preparation of high-performance Cu-1 wt%Ag wires; in addition, the study of the cold deformation is of great significance for the prediction of future changes in the properties of alloy wires under the same drawing conditions.

### 3.5. Effect of Different Heat Treatment Temperatures on the Microstructure of Cu-1 wt%Ag Wire

Higher heat treatment temperature helps grain growth, while lower temperature is conducive to grain refinement. The annealing temperature range was set to 250–550 °C in order to explore the influence of different temperatures on the microstructure and properties of alloy wire in the heat treatment process, to ensure that the recrystallization phenomenon of the alloy wire can fully occur, and to find the temperature range of the occurrence of the recrystallization phenomenon.

**Figure 8:**
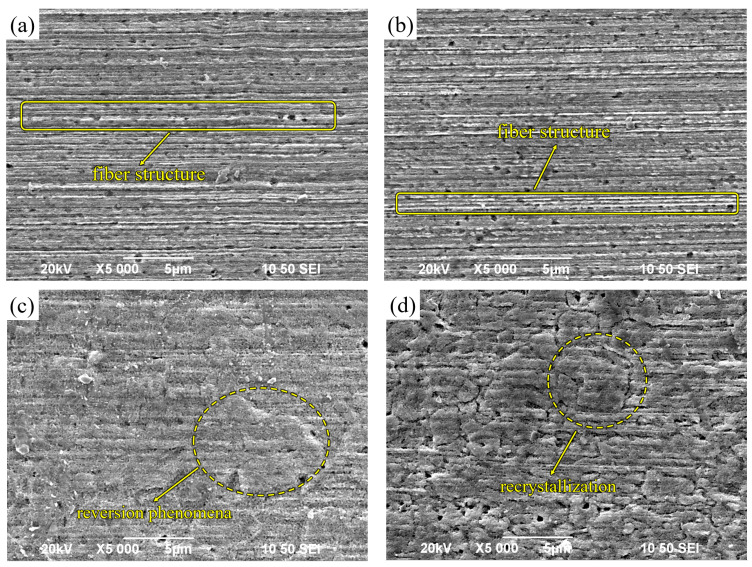
Longitudinal microstructure of Cu−1 wt% Ag wires annealed at different temperatures ((**a**) unannealed; (**b**) 400 °C; (**c**) 500 °C; (**d**) 550 °C).

Figure 8a illustrates the microstructure of unannealed Cu-1 wt%Ag wires. At this stage, the Cu-1 wt%Ag wire exhibits a fibrous structure, with the diameter of the fibers being noticeably smaller at the wire’s edges compared to its center. This variation in diameter is primarily caused by the uneven distribution of forces during the drawing process. The edge portion of the wire comes into contact with the die first and experiences both shear deformation and extrusion from the die simultaneously. As a result, the deformation in the edge region is more severe compared to other areas of the wire.

Figure 8b presents the microstructure of Cu-1 wt%Ag wire in the annealed state at 400 °C. The addition of Ag raises the recrystallization temperature, which causes the Cu-1 wt%Ag wire to begin undergoing reversion. As a result, the dislocation density decreases. However, due to the relatively low heat treatment temperature, a significant number of deformed regions still exist within the wire. These regions exhibit a fibrous structure aligned along the drawing direction.

Figure 8c displays the microstructure of Cu-1 wt%Ag wire after annealing at 500 °C. With increasing annealing temperature, the wire exhibits enhanced recovery, resulting in the release of distortion energy and the precipitation of a secondary Ag phase in close proximity to the fiber phase. Simultaneously, the elevated temperature induces intensified movement of dislocations, vacancies, and other lattice defects. These defects are gradually absorbed by a significant number of internal stresses, leading to their gradual release. Consequently, the deformation structure gradually disappears, initiating the process of recrystallization.

Figure 8d illustrates the microstructure of Cu-1 wt%Ag wire in the annealed state at 550 °C. Subsequent to annealing at this temperature, there is an enhanced propensity for the release of distortion energy, leading to intensified movement of dislocations and vacancies with a further reduction in their density. During this stage, recrystallization initiates in the Cu-1 wt%Ag wire, resulting in gradual grain growth. The microstructure exhibits diminished visibility of the deformation organization, and some coarsened grains become apparent. As the level of recrystallization progresses, phase interfaces undergo complete activation, and there is pronounced migration of grain boundaries. Consequently, the fiber structure tends to vanish and redistribute, facilitating the growth of grains.

### 3.6. Effect of Different Heat Treatment Temperatures on the Properties of Cu-1 wt%Ag Wire

**Figure 9:**
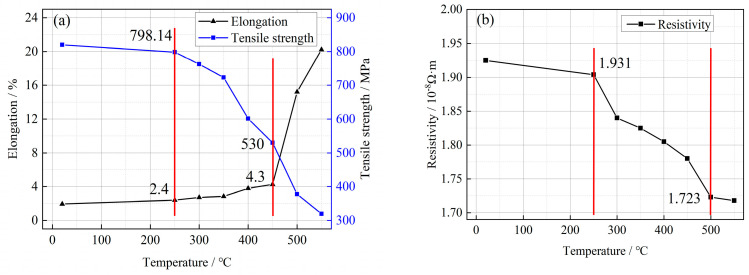
Mechanical and electrical properties of Cu−1 wt%Ag wires at different annealing temperatures ((**a**) mechanical properties; (**b**) electrical properties).

Figure 9a shows the variation of mechanical properties of 0.08 mm diameter Cu-1 wt%Ag bonded wires at different heat treatment temperatures. The unannealed Cu-1 wt%Ag bonded wire exhibited high tensile strength of 820.1 MPa and low elongation of 1.94%. Following annealing at 250 °C, the Cu-1 wt%Ag wire displayed a slight change in mechanical properties, with a tensile strength of 798.14 MPa and elongation of 2.4%. As the annealing temperature increased from 300 °C to 350 °C, significant changes in the properties of the Cu-1 wt%Ag wire were observed compared to the initial sample. These changes included a decrease in tensile strength to 723 MPa and an increase in elongation to 2.84%. Further increasing the annealing temperature, the tensile strength of the Cu-1 wt%Ag wire decreased by 197.1 MPa, reaching 530 MPa after annealing at 450 °C. Additionally, the elongation increased to 4.3%. Continuing to raise the annealing temperature resulted in further property changes in the Cu-1 wt%Ag wire. Compared to the sample annealed at 450 °C, the tensile strength decreased by 210 MPa to 319.5 MPa when annealed at 550 °C, while the elongation significantly increased to 20.2%.

In the Cu-1 wt%Ag wire, after undergoing significant deformation, the cross-sectional area experiences a rapid reduction. This reduction leads to an increase in the density of grain boundaries per unit cross-sectional area. Additionally, an abundance of vacancies, dislocations, and other defects accumulate at the grain boundaries, forming an isolation layer rich in crystalline defects. As a result, the electron scattering effect is enhanced within the wire.

In Figure 9b, the electrical properties of a Cu-1 wt%Ag bonding wire with a diameter of 0.08 mm are shown at different heat treatment temperatures. The unannealed Cu-1 wt%Ag wire exhibits a higher resistivity of 1.931 × 10^−8^ Ω·m. This increased resistivity is attributed to the presence of defects such as dislocations and vacancies within the material. When the wire is subjected to heat treatment below 250 °C, the resistivity decreases slightly to 1.904 × 10^−8^ Ω·m. At this temperature, the microstructure of the wire does not undergo significant changes compared to its unannealed state. As the annealing temperature is increased to 300 °C, intense movement of point defects, such as vacancies and interstitial atoms, takes place. Vacancies migrate towards the grain boundaries and neutralize with interstitial atoms. This results in a decrease in the density of point defects, leading to a decrease in resistivity to 1.840 × 10^−8^ Ω·m. Between 300 °C and 450 °C, the dislocation density gradually decreases, and the fibrous structure begins to disappear. This reduction in dislocation density and fibrous structure contributes to a further decrease in resistivity. The resistivity of the wire decreases to 1.78 × 10^−8^ Ω·m within this temperature range. As the annealing temperature continues to increase, the reversion effect becomes more prominent, leading to the disappearance of the fibrous structure in large quantities. Recrystallization of the material initiates, albeit at a slow rate. As a result, the ability of the material to scatter electrons decreases. The resistivity of the wire continues to decrease, and after annealing at 550 °C it reaches 1.718 × 10^−8^ Ω·m.

In the heat treatment test at different temperatures, the annealing temperature below 250 °C, the microstructure and properties of alloy wires have no obvious changes, but the value of tensile strength and resistivity has a tendency to decline, and the value of the elongation has a tendency to increase, although the annealing temperature is low, but the alloy wires also have a certain performance impact; When the annealing temperature is 250–500 °C, the performance of the alloy wire has the same change trend as that of low annealing temperature, and the change is more obvious. This suggests that lower annealing temperatures may cause a slight change in properties, while annealing at temperatures in the range of 250–500 °C may change the properties of the alloy wires more significantly.

### 3.7. Effect of Different Annealing Rates on the Properties of Cu-1 wt%Ag Wires

Lower annealing rates usually increase the toughness and ductility of the material but may decrease the hardness and strength. Higher annealing rates may increase hardness and strength but may decrease toughness. The annealing rate is set to control the time of annealing, to study the performance of alloy wires at different annealing rates, and to find the annealing rate parameter for the excellent performance of alloy wires in conjunction with experimental data.

**Figure 10:**
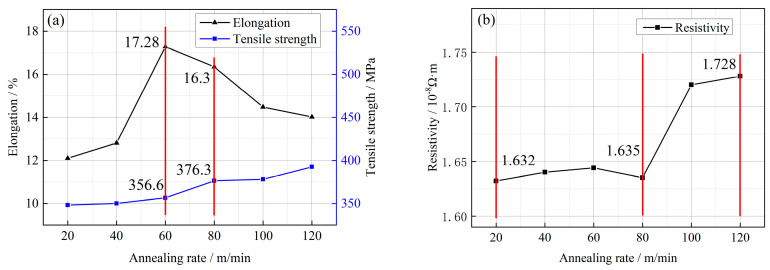
Mechanical and electrical properties of Cu−1 wt%Ag wires at different annealing rates ((**a**) mechanical properties; (**b**) electrical properties).

Figure 10a illustrates the impact of different heat treatment rates on the mechanical properties of Cu-1 wt%Ag bonded wires. For annealing rates ranging from 20 to 120 m/min, the tensile strength of the Cu-1 wt%Ag wires gradually increases from 348.3 MPa to 392.7 MPa. This increase in tensile strength is relatively slow within this annealing rate range. At annealing rates between 80 and 120 m/min, the wires are in the recovery stage. During this stage, the internal work hardening caused by the drawing process is gradually eliminated, and the degree of recovery becomes more pronounced as the annealing rate decreases. As a result, the elongation of the wires increases from 14% (at 120 m/min) to 16.3% (at 80 m/min). When the annealing rate is 60 m/min, the material reaches its peak elongation of 17.28%. At this point, the material has fully reverted internally and starts to recrystallize. However, at an annealing rate of 20 m/min, the elongation decreases to 12.1%. This reduction in elongation is attributed to the slow annealing rate, which leads to severe grain coarsening in the material.

Figure 10b presents the changes in resistivity of Cu-1 wt%Ag bonded wire for different heat treatment rates. With a decrease in annealing rate, the overall resistivity of Cu-1 wt%Ag wire tends to decrease. At an annealing rate of 120 m/min, the resistivity of Cu-1 wt%Ag wires is relatively high at 1.728 × 10^−8^ Ω·m. This is because the fast annealing rate does not allow sufficient time for the wires to fully recover. As the annealing rate decreases, the resistivity of the material gradually reduces. At an annealing rate of 20 m/min, the resistivity reaches a lower value of 1.632 × 10^−8^ Ω·m. This slower annealing rate allows for improved recovery and leads to a decrease in resistivity.

## 4. Conclusions

(1) After a single-pass drawing with deformation amounts of 18%, 14%, and 10%, the grains gradually transformed into a fibrous morphology, and Ag fiber grain organization was observed within the Cu-1 wt%Ag wires. The addition of the Ag element increased the deformation resistance of the wires. Specifically, the tensile strength of the Cu-1 wt%Ag wires increased from 670 MPa to 784.63 MPa, 783.9 MPa, and 782.58 MPa for deformation amounts of 18%, 14%, and 10%, respectively. Additionally, the resistivity increased from 1.922 × 10^−8^ Ω·m to 1.959 × 10^−8^ Ω·m, 1.923 × 10^−8^ Ω·m, and 1.923 × 10^−8^ Ω·m, respectively. Overall, the wires exhibit superior performance when subjected to a single-pass deformation of 14%.
(2) Following the experiments conducted with various drawing rates, the tensile strength of Cu-1 wt%Ag wires increased from 783.9 MPa to 806 MPa, 820.1 MPa, and 817.1 MPa, respectively. Meanwhile, the electrical resistivity rose from 1.923 × 10^−8^ Ω·m to 1.927 × 10^−8^ Ω·m, 1.931 × 10^−8^ Ω·m, and 1.954 × 10^−8^ Ω·m, respectively. Notably, when employing the drawing rate of 300 m/min, the resulting wires consistently exhibited low resistivity and showcased smooth performance transitions throughout the drawing process.
(3) The resistivity of the Cu-1 wt%Ag wires exhibited minimal changes prior to annealing temperatures of 250 °C. At an annealing temperature of 300 °C, the Cu-1 wt%Ag bonded wires commenced reversion, with a more pronounced effect observed above 350 °C. At 450 °C, the resistivity of the Cu-1 wt%Ag wire decreased to 1.78 × 10^−8^ Ω·m. Further increasing the temperature to 550 °C resulted in a substantial reduction in the fiber phase, accompanied by gradual recrystallization of the material. However, the recrystallization rate was relatively slow, leading to a continued decrease in the material’s overall ability to scatter electrons. Consequently, the resistivity steadily declined, reaching a final value of 1.718 × 10^−8^ Ω·m. The Cu-1 wt%Ag wires exhibited enhanced performance following annealing between 500 °C and 550 °C.
(4) Within the annealing rate range of 20 to 120 m/min, the tensile strength of Cu-1 wt%Ag wires exhibited a gradual increase, ranging from 348.3 MPa to 392.7 MPa. At an annealing rate of 60 m/min, the material underwent complete internal reversion and initiated recrystallization, resulting in a peak elongation of 17.28%. With a decrease in the annealing rate to 20 m/min, the recrystallized grains began to grow, leading to a significant reduction in elongation by 12.1% compared to the 60 m/min annealing rate. Concurrently, the resistivity of the Cu-1 wt%Ag wire increased from 1.632 × 10^−8^ Ω·m (at 20 m/min) to 1.728 × 10^−8^ Ω·m (at 120 m/min). Based on these findings, the optimal annealing rate for Cu-1 wt%Ag wire falls within the range of 60–80 m/min.
(5) For the cold deformation test, different drawing process parameters lead to different performance, and the variation of drawing rate with microstructure is not obvious, suggesting that the setting of the drawing rate is not comprehensive enough. The drawing rate interval is small, not exceeding 700 m/min or falling below 300 m/min in the study. At high and low drawing rate, the microstructure should have obvious differences. In heat treatment experiments, the addition of Ag elements of copper wire, compared with pure copper recrystallization phenomenon, occurs at a higher temperature, and the consideration is that a stronger chemical affinity between silver and copper leads to the formation of a more stable interface, increasing the temperature required for recrystallization.

## Figures and Tables

**Table 1:** Mainly used equipment.

Equipment	Model
Fine Wire-Drawing Machine	LH160-24
Ultra-Fine Wire-Drawing Machine	LH150-36
Scanning Electron Microscope	JEOL JSM-6700F
Tensile Testing Machine	HS-3004A
Microcomputer-Controlled Electronic Universal Tester	KD2-0.02
Resistance Tester	SB2231
Heat Treatment Equipment	SD-40

**Table 2:** Variation of wire strain in the drawing tests.

Test Type	Wire Diameter (mm)	Drawing Strain
Different deformation amount test	0.25–0.135	0–1.232
Different drawing rate test	0.135–0.08	0–1.046

**Table 3:** Drawing tests with different deformation.

Program Number	Initial Wire Diameter (mm)	Single-Pass Reduced Surface Volume	Number of Molds	Drawing Speed (m/min)	Final Wire Diameter (mm)
Program 1	0.25	18%	7	500	0.135
Program 2		14%	9		
Program 3		10%	12		

**Table 4:** Different drawing rate tests.

Program Number	Initial Wire Diameter (mm)	Single-Pass Reduced Surface Volume	Drawing Speed (m/min)	Final Wire Diameter (mm)
Program 1	0.135		300	0.08
Program 2		15%	500	
Program 3			700	

**Table 5:** Different annealing process tests.

Test Type	Wire Diameter (mm)	Annealing Temperature (°C)	Annealing Rate (m/min)
Different annealing temperature	0.08	250–500	100
Different annealing rate		500	20–120

## Data Availability

Sorry, since the partner needs to keep the original data confidential, it cannot be shared.
